# Enhanced Degradation of Sulfamethoxazole (SMX) in Toilet Wastewater by Photo-Fenton Reactive Membrane Filtration

**DOI:** 10.3390/nano10010180

**Published:** 2020-01-20

**Authors:** Shaobin Sun, Hong Yao, Xinyang Li, Shihai Deng, Shenlong Zhao, Wen Zhang

**Affiliations:** 1 Beijing International Scientific and Technological Cooperation Base of Water Pollution Control Techniques for Antibiotics and Resistance Genes, Beijing Key Laboratory of Aqueous Typical Pollutants Control and Water Quality Safeguard, Department of Municipal and Environmental Engineering, School of Civil Engineering, Beijing Jiaotong University, Beijing 100044, China; ssbzwj@163.com (S.S.); lixinyang@bjtu.edu.cn (X.L.); ceedeng@nus.edu.sg (S.D.); Shenlong.zhao@sydney.edu.au (S.Z.); wen.zhang@njit.edu (W.Z.); 2 John A. Reif, Jr. Department of Civil and Environmental Engineering, New Jersey Institute of Technology, Newark, NJ 07102, USA; 3 Department of Civil and Environmental Engineering, National University of Singapore, 10 Kent Ridge Crescent, Singapore 119260, Singapore; 4 School of Chemical and Biomolecular Engineering, The University of Sydney, Sydney 2006, Australia; 5 School of Environmental and Municipal Engineering, Qingdao University of Technology, Qingdao 266033, China

**Keywords:** photo-fenton, ceramic membrane, toilet wastewater, SMX, α-FeOOH

## Abstract

Pharmaceutical residuals are increasingly detected in natural waters, which made great threat to the health of the public. This study evaluated the utility of the photo-Fenton ceramic membrane filtration toward the removal and degradation of sulfamethoxazole (SMX) as a model recalcitrant micropollutant. The photo-Fenton catalyst Goethite (α-FeOOH) was coated on planar ceramic membranes as we reported previously. The removal of SMX in both simulated and real toilet wastewater were assessed by filtering the feed solutions with/without H_2_O_2_ and UV irradiation. The SMX degradation rate reached 87% and 92% respectively in the presence of UV/H_2_O_2_ for the original toilet wastewater (0.8 ± 0.05 ppb) and toilet wastewater with a spiked SMX concentration of 100 ppb. The mineralization and degradation by-products were both assessed under different degradation conditions to achieve deeper insight into the degradation mechanisms during this photo-Fenton reactive membrane filtration. Results showed that a negligible removal rate (e.g., 3%) of SMX was obtained when only filtering the feed solution through uncoated or catalyst-coated membranes. However, the removal rates of SMX were significantly increased to 67% (no H_2_O_2_) and 90% (with H_2_O_2_) under UV irradiation, respectively, confirming that photo-Fenton reactions played the key role in the degradation/mineralization process. The highest apparent quantum yield (AQY) reached up to approximately 27% when the H_2_O_2_ was 10 mmol·L^−1^ and UV254 intensity was 100 μW·cm^−2^. This study lays the groundwork for reactive membrane filtration to tackle the issues from micropollution.

## 1. Introduction

Pharmaceutical residuals are increasingly detected in natural waters and effluent from wastewater treatment plants (WWTPs) [1]. This raises public health concerns even though most of the detected pharmaceuticals in the environment are at low sub-therapeutic concentrations (e.g., 110~610 ng∙L^−1^) [2,3]. Pharmaceutical residuals may be released from various sources such as disposed medicines [4], urine [5], contaminaed soil [6], and industrial wastewater [7]. Among these sources, human urine releases pharmaceuticals with concentrations at 2–3 orders of magnitude higher than other municipal wastewater streams that enter WWTPs [8]. Pharmaceuticals such as sulfamethoxazole (SMX) are commonly prescribed to treat infectious and respiration diseases. Sulfonamide antibiotics are discharged in feces and urine, either as parent compound or metabolites [9].

Different treatment processes have been investigated for removing pharmaceuticals from urine. These processes range from membrane filtration [10], anion exchange resin [11,12], electrodialysis [12], and struvite precipitation [13]. Most of the treatment processes results in separation, concentration, and fixation of water pollutants in other forms such as solid waste. Additional chemical destruction of the sulfonamides and other pharmaceuticals are often necessary for avoiding secondary pollution [14]. Advanced oxidation processes (AOPs) such as UV/O_3_, UV/H_2_O_2_, photocatalysis, ozonation, electrochemical oxidation, Fenton, and Fenton-like processes are commonly used to mineralize diverse recalcitrant organic pollutants including pharmaceutical pollutants [15,16,17]. Moreover, combination of AOPs and membrane filtration has gained a great deal of attention recently as AOPs show synergistic roles in membrane filtration by enhancing pollutant degradation [18,19], improving filtration performance [20], and mitigating membrane fouling [21,22,23]. For example, our previous work [24,25] and a few others [26,27], heterogeneous photo-Fenton reaction was coupled with ceramic membrane filtration and increased the removal efficiency of 55 ± 5% and surface foulants (e.g., BSA and humic acid). Yang Guo developed a novel catalytic ceramic membrane with a coating layer of CuMn_2_O_4_ particles that increased ozonation and filtration performances. The modified membrane increased the additional removal rate of benzophenone-3 from 28% to 34% and reduced the toxicity of degradation intermediates with a drop of EC_50_ by 12.77% [28]. This improvement was ascribed to the surface-catalytic reactions between ozone and CuMn_2_O_4_ particles that enhanced the ozone self-decompose to generate. Other reactive membrane systems (e.g., microwave-enhanced membrane filtration [29], photocatalytic ceramic membrane [24,25,30,31,32], and electrochemical ceramic membrane [33,34,35,36]) were recently tested for their removal capabilities of various micropollutants including 1,4-dioxane, dyes, and drugs.

This study evaluated the utility of the photo-Fenton ceramic membrane filtration toward the removal and degradation of SMX as a model recalcitrant micropollutant. Goethite (α-FeOOH) was coated on planar ceramic membranes as the photo-Fenton catalyst as we reported previously [37]. The removals of SMX were assessed not only in simulated feed water but also the real toilet wastewater, which were filtered through the coated membrane with/without H_2_O_2_ and UV irradiation. The TOC removal and degradation by-products were both assessed under different degradation conditions to achieve deeper insight into the degradation mechanisms during this photo-Fenton reactive membrane filtration.

## 2. Material and Method

### 2.1. Functionalization of Ceramic Membrane

Synthesis of α-FeOOH catalysts and surface coating on the flat-sheet ceramic membrane (47M014, Sterlitech Ceramic Membrane, Kent, WA, USA) were conducted following the reported methods [24]. Briefly, α-FeOOH catalysts were synthesized in a precipitation method, where 0.5 mol·L^−1^ Fe(NO_3_)_3_ was titrated with a 2.5 mol·L^−1^ NaOH solution until the reaction solution reached a pH of 12. Then, the suspension was oven dried at 60 °C for 12 h and cooled at room temperature. The precipitate washed repeatedly with DI water and vacuum dried at 60 °C for 2 h. The synthesized catalyst particles with a length of approximately 400–500 nm and the width of about 25–50 nm were immobilized onto planar membranes using Bis-(3-[triethoxysilyl]-propyl)-tetrasulfide (22.3%, w/w, in water) as a silane binder. The coating density, approximately 2 µg-catalyst g^−1^-membrane, was consistently used in most of the experiment below. Moreover, the coating density was varied by depositing different amounts of catalyst on membranes to examine the impact of coating structures on permeate flux. The catalyst surface coverage and thickness were examined by SEM.

### 2.2. Batch Degradation Experiments under Different Conditions

To examine the contributions of membrane adsorption, UV photolysis and photo-Fenton reaction toward the removal of SMX, a series of bath experiments were carried out with/without UV_254_ irradiation, H_2_O_2_, and the presence of α-FeOOH catalyst on the membrane. Briefly, 30 mL of the SMX solution with the initial concentration of 12 mg·L^−1^ was prepared. Then, the ceramic membrane (47 mm in diameter and 2.5 mm in thickness) with or without the coating of α-FeOOH was placed on the bottom of the 90-mm petri dish as shown in Figure S1 in the Supporting Information (SI). The distance between the UV lamp and the surface of the liquid was 2.5 cm to obtain approximately the exposure intensity of 400 ± 1 µW∙cm^−2^. The petri dish was mildly agitated on a rotational shaker to thoroughly mix up the solution. The dose of H_2_O_2_ was consistently 10 mM for all the experiments unless indicated. 0.5 mL samples were taken at different times (0, 1, 5, 10, 20, 30, and 60 min) and filtered before the analytical measurement of the SMX concentrations by a high-performance liquid chromatography (HPLC, WATERS e2695, USA) as detailed in the Supporting Information.

### 2.3. Filtration Experiments

#### 2.3.1. Operation of Continuous Filtration Experiments

The removal and degradation of SMX were also assessed in a dead-end mode filtration through the catalyst-coated ceramic membrane. The membrane filtration module was made of polytetrafluoroethylene (PTFE) that is highly resistant to chemical oxidation or UV irradiation. The available membrane surface area was approximately 17.34 cm^2^ with an overhead space of 1.9 ml (0.2 cm in depth) and a quartz window allowing the UV light illumination (Figure S2). A UVL 214-Watt lamp (Analytikjena Company, Beverly, MA, USA) provides a monochromatic UV_254_ irradiation of 401 μW·cm^−2^ on the surface of the α-FeOOH-coated membrane. More detailed illustration of the photocatalytic membrane filtration was reported previously [24,25].

#### 2.3.2. Degradation of SMX Spiked in Toilet Wastewater via Photocatalytic Membrane Filtration

Real toilet water was taken from railway stations in China and filtrated by mixed cellulose ester (MCE) membranes with a nominal pore size 0.45 µm. The background SMX concentration was determined to be 0.8 ± 0.05 ppb. To accurately assess the removal of SMX in the real toilet water, SMX was spiked to reach a final concentration of 100 ppb in the tested water by adding 0.1 mL of the SMX stock solution (1 mg·L^−1^) to 1 L of the real toilet water that was pre-filtrated with 0.45-μm glass fiber membrane filters. The pH of the real toilet water varied slightly (5.7–7.3), which was adjusted to 7 with phosphate buffer or NaOH. Other major real toilet water quality parameters are shown in Table 1.

### 2.4. Analysis of Photocatalytic Degradation Mechanisms

To examine the photocatalytic degradation pathways for SMX, influent and effluent samples from the photo-Fenton reactions were analyzed for degradation byproducts using liquid chromatography−electrospray ionization mass spectrometry equipped with an electrospray ionization source (ESI) or LC−ESI−MS (Agilent1290-6430, USA).

### 2.5. Statistical Analysis

**Figure 1:**
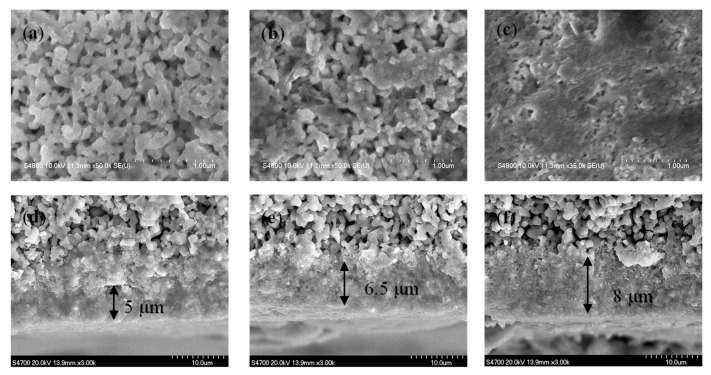
SEM images of α-FeOOH coated ceramic membranes. (**a**–**c**) Top views for ceramic membranes with low coating, medium coating and heavy coating. (**d**–**f**) Cross-sectional views for ceramic membranes with low coating, medium coating, and heavy coating corresponding to coating densities of 0.5, 2 and 6 µg-catalyst·g-membrane^−1^.

**Figure 2:**
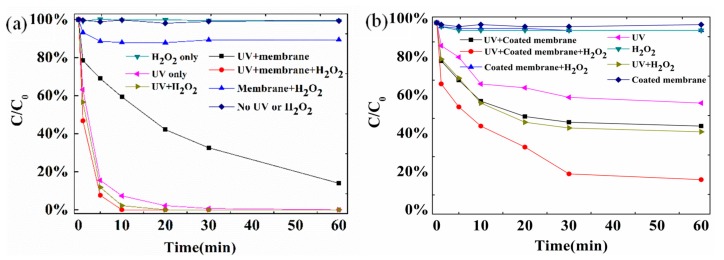
(**a**) The ratio of the remaining concentration (C) of SMX over the initial concentration (C_0_) under different degradation processes on catalyst-coated ceramic membrane. (**b**) The TOC removal in the batch photo-Fenton reactions with or without the presence of the catalyst-coated ceramic membrane. Initial SMX concentration: 20 mg·L^−1^ corresponding to an initial TOC concentration of 5.8 mg·L^−1^, UV wavelength was 254 nm and intensity was 401 µw·cm^−2^; H_2_O_2_ concentration was 10 mmol·L^−1^, and the catalyst on the ceramic membrane was 2 μg·g^−1^. The doses of UV irradiation and H_2_O_2_ on SMX photodegradation were optimized with details discussed in Section S4.

**Figure 3:**
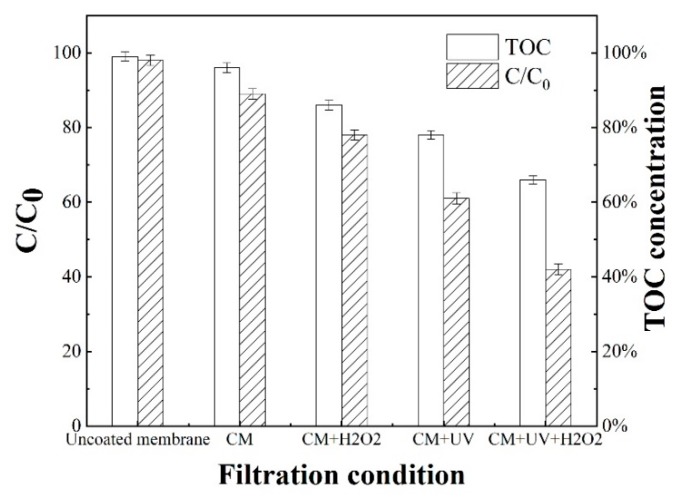
The removal of SMX under different filtration conditions: SMX concentration: 20 mg·L^−1^; Influent flux: 10 LMH; UV intensity: 401 μW·cm^−2^; H_2_O_2_ dosage: 10 mmol·L^−1^ at 5 ± 0.2 μL∙s^−1^ and CM denotes for coated membrane.

**Figure 4:**
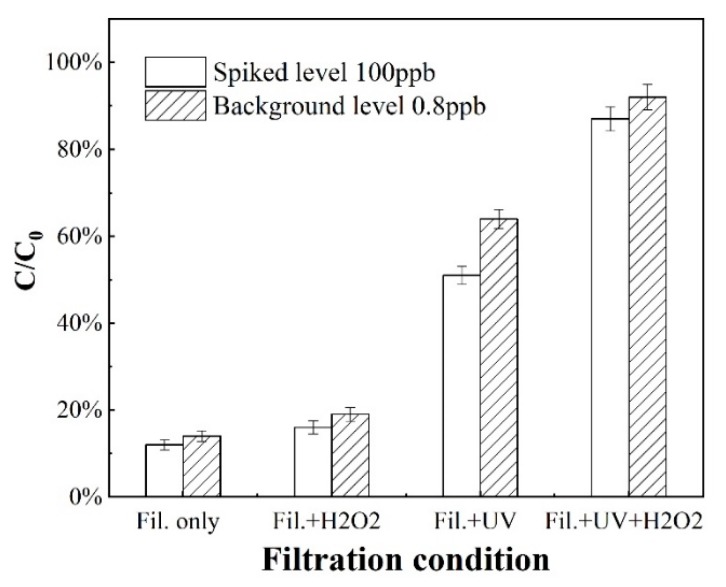
The SMX removal in initial and spiked toilet water under different degradation processes on catalyst-coated ceramic membrane. The background SMX concentration: 0.8 ± 0.05 ppb, spiked SMX concentration: 100 ppb. The UV_254_ intensity was 401 µW·cm^−2^; H_2_O_2_ concentration was 10 mmol·L^−1^; and the catalyst on the ceramic membrane was 2 µg·g^−1^. “Fil.” stands for filtration with a permeate flux of 10 LMH.

**Figure 5:**
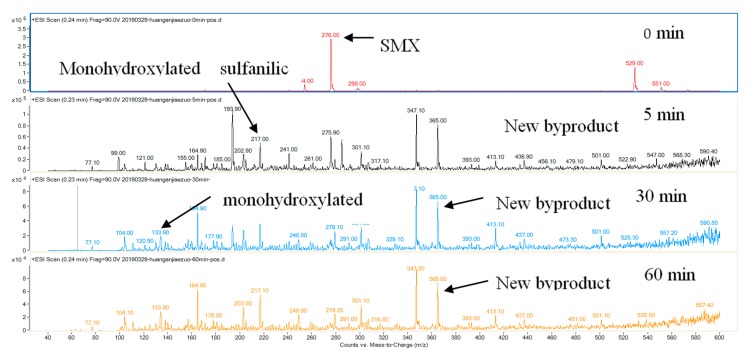
The RRLC-MS analysis of SMX and its degradation byproducts in the liquid samples that underwent different reaction times (0, 5, 30, and 60 min).

**Figure 6:**
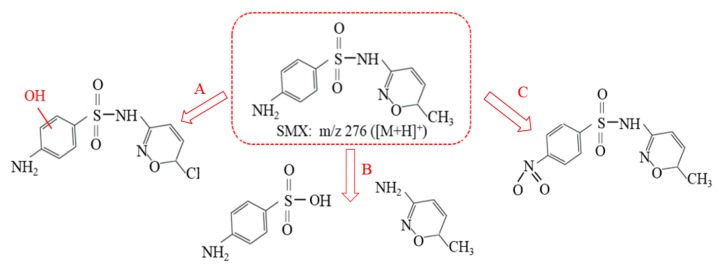
Reaction pathways of SMX and the major intermediates observed in this study.

The following experiments were carried out at least with triplicate independent sampling or testing: (1) DI water permeation test; (2) degradation assessment of SMX in batch mode; (3) the concentration measurement of SMX and TOC. SEM images in Figure 1 are typical results selected from at least five sample locations, while the presented results in Figure 2, Figure 3 and Figure 4 are usually presented with average values with standard deviation as error bars. For the filtration studies, permeate samples were taken at multiple sampling times to obtain representative results, which were shown as average (Figure 5 and Figure 6). However, three repetitions of filtration tests were conducted to confirm the observations. *t*-testing was used to examine the significance of data variations we observed in Figure 5 in different filtration conditions using at a significant level of 0.05.

## 3. Results and Discussion

### 3.1. Catalyst Coating Density and Impacts of Membrane Permeability

Figure 1 shows the SEM images of the coated membranes from the top and cross-sectional views. As the catalyst coating density increased from 0.5 to 6 µg·g^−1^, the surface coverage apparently increased, and the resulting pores seemed to decrease as compared between Figure 1a,c. The cross-sectional images show that the coating thickness varied from 5 to 8 µm accordingly. Figure S3 shows the water permeability under various TMPs for ceramic membranes before and after catalyst coating. The permeate flux (L m^−2^ h^−1^, LMH) was calculated by the Darcy’s equation under different TMPs as detailed in Section S3. The water permeability for the pristine ceramic membranes with a nominal pore diameter of 0.14 μm are determined to be more than 44.0 LMH∙psi^−1^. For coated membranes, the water permeability reduced to 10, 13, and 20 LMH∙psi^−1^ under heavy coating, medium coating and low coating. The inherent membrane resistance (*R_m_*) for the pristine ceramic membranes was 0.8 × 10^10^ m^−1^. With the catalyst coating, the values of *R_m_* increased to 3.45 × 10^10^ m^−1^, 2.74 × 10^10^ m^−1^, and 1.98 × 10^10^ m^−1^ under heavy coating, medium coating, and low coating conditions.

### 3.2. Assessment of Pollutant Degradation in Batch Experiments

Figure 2 compares the degradation rates of SMX on catalyst-coated membranes under different conditions. The removal rate of SMX was negligible when the SMX solution was only exposed to catalyst-coated membrane, implying that the surface adsorption of SMX on coated membrane was minor. Similarly, the SMX degradation was also negligible if only H_2_O_2_ was present in the solution. When the membrane was present with addition of H_2_O_2_, the SMX removal slightly increased to a stable level of over 5% after 10 min. By contrast, the SMX removal was significantly improved under UV irradiation, which alone led to a progressive SMX degradation as shown by the purple triangle data. With the combination with UV/H_2_O_2_ or the coated membrane/UV/H_2_O_2_, the SMX removal efficiencies were substantially increased. UV irradiation alone appeared to cause SMX degradation or photolysis, especially in the presence of the catalyst-coated membranes, on which UV photocatalytic reactions may occur. The results in Figure 2a were fitted using a first-order degradation kinetics [38]. The corresponding rate constants (*k*) and the squared correlation coefficients (*R*^2^) are summarized in Table 2. The highest reaction rate constant was obtained when using the coated membrane under UV/H_2_O_2_, confirming that photo-Fenton reaction on the membrane was the primary factor for the enhanced degradation of SMX [24,25].

Figure 2b shows the TOC changes in the SMX solution under different reaction conditions. No mineralization of SMX was obtained when the solution was exposed to the catalyst-coated membrane or H_2_O_2_ only. By contrast, a TOC removal rate of 90% at 60 min when UV/H_2_O_2_ were both applied to the catalyst-coated membrane. However, the mineralization of SMX was reduced to 49% and 42% at 20 min if the solution was only exposed to UV/H_2_O_2_ or to the catalyst-coated membrane under UV irradiation only.

### 3.3. Pollutant Removal and Degradation in Continuous Filtration

#### 3.3.1. Removal of SMX under Different Membrane Filtration Conditions

Figure 3 shows that approximately 2% of SMX was removed by the uncoated membrane with less than 1% of TOC reduction, which indicates that the contributions from the size exclusion or membrane surface adsorption for SMX are negligible. Meanwhile, filtration through the catalyst-coated membrane slightly increased the removal rate of SMX to about 11%. By contrast, when the catalyst-coated membrane was only exposed to H_2_O_2_, 20% of SMX and 13% of TOC were was removed, indicating that the degradation of SMX was slightly enhanced but the mineralization was still minor. In the presence of catalyst on the membrane and UV irradiation, both the removal rates of SMX and TOC were significantly increased to 40% and 22% respectively, which agrees with the results from the batch experiments. Furthermore, when applying UV irradiation and H_2_O_2_ onto the catalyst-coated membrane, the removal of SMX reached the highest level (almost 58%), confirming that the degradation/mineralization was primarily attributed to the photo-Fenton reactions.

#### 3.3.2. Assessment of SMX Removal in Toilet Wastewater

Figure 4 compares the degradation rates of SMX in toilet wastewater with/without spiked SMX on catalyst-coated membranes under different filtration conditions. Clearly, the degradation rates of SMX in the real toilet water well align with in the spiked toilet water, though the SMX removal rates were higher in the spiked toilet water than in the real toilet water. The removal rate of SMX was negligible when the SMX solution was filtrated only by adding H_2_O_2_ to the feed solution. By contrast, the SMX removal was significantly improved under UV irradiation, which alone led to a progressive SMX degradation to 50% and 60% in raw and spiked toilet wastewater respectively. With the combination with UV/H_2_O_2_, the SMX removal efficiencies were substantially increased to 87% and 92% for the raw and spiked toilet wastewater respectively.

Apparently, the background constituents in the raw toilet wastewater such as dissolved organic matters with a TOC of 1712 ± 18 mg·L^−1^, suspended solids (983 ± 9 mg·L^−1^) and ammonia nitrogen (1218 ± 16 mg·L^−1^) did not negatively affect the photodegradation of SMX on the ceramic membrane surface, although these species may potential sequester and consume photogenerated radicals. This is probably because the operating photo-Fenton reaction exhibited a greater electron scavenging or transferring rates than the level imposed from the filtering wastewater. The rate (e^−^∙s^−1^) of electrons (*J_P_*) transferred from valence band to the conduction band on photocatalyst can be calculated by

*J_P_* = *η* × UV intensity × surface area/band gap (1)

where *η* is the apparent quantum yield (e.g., 5–15%), the UV intensity was 400 µW∙cm^−2^, the effective UV-exposure area was about 12.56 cm^2^ and the band gap of FeOOH catalyst was 2.5 eV (1 eV = 1.6 × 10^−19^ *J*). The total electron loading rate (*J_e_*) from the influent is a function of the flow rate (*Q*) and concentrations of equivalent electrons

(2) $$J_{e}=Q\cdot \sum_{i}n_{i}C_{i}$$

where *i* refers to the electron donor species (SMX, TOC, and NH_4_^+^-N); *n* is the number of the total electrons from electron donor species (*e*^−^ mole^−1^); (e.g., for SMX, *n* = 42 *e*^−^∙mole^−1^, for TOC, *n* was taken as 8 *e*^−^ mole^−1^; and for NH_4_^+^-N, *n* = 8 *e*^−^ mole^−1^ if completely oxidized to nitrate); *C_i_* is the concentration of electron donor species (mg·L^−1^ or mol·L^−1^); and Q is the feed flow rate (3.5 µL∙s^−1^). Applying our filtration and experimental conditions, we determined that *J_e_* (5.89 × 10^−6^ e^−^∙s^−1^) was much smaller than *J_P_* (1.26 ± 0.63 × 10^15^ e^−^·s^−1^, assuming *η* = 15%), which explains why the background pollutants did not significantly affect the removal rates of SMX. This result also agrees with the previous studies showing that TOC and ammonia could be efficiently oxidized by photo-Fenton reactions [39,40].

### 3.4. Analysis of Photocatalytic Degradation Mechanisms

The surface sites (≡FeIII(OH)) on α-FeOOH catalyst are considered to catalyze the generation of hydroxyl radicals and peroxide anions via photo-Fenton reactions [24]. As reported previously [41], ·OH stoichiometricaly reacts with *p*-Chlorobenzoic acid (*p*CBA) in a mole ratio of 1:1, and degradation of ·H through side reactions with other potential contaminants in our reaction system can be ignored. Our results show that UV or H_2_O_2_ alone did not decrease the *p*CBA concentration significantly, which similarly occurred to coated membrane with or without the addition of H_2_O_2_. When exposed to UV alone or the catalyst-coated membrane, the *p*CBA concentration started to decline much faster. Furthermore, when UV/H_2_O_2_ was applied with or with the catalyst-coated membrane, the *p*CBA concentration declined sharply, indicative of the generation of OH via photocatalysis or photo-Fenton reactions. The detailed measurement of *p*CBA has been reported elsewhere [24].

To gain a better understanding of mineralization mechanisms, the oxidation byproducts of SMX were identified by LC−ESI−MS. Figure 5 shows the chromatograms of the treated permeate samples at different reaction times (0, 5, 30, and 60 min). Four main oxidation intermediates and SMX are identified based on their characteristic peaks at different *m*/*z* values. Figure 6 illustrates the hypothetical degradation or transformation pathways (A, B, and C) of SMX in the photo-Fenton oxidation reaction. In pathway A, the SMX forms monohydroxylated sulfamethoxazole by direct attack of HO· on the aromatic moiety of SMX and/or hydrolysis of unstable radical cation SMX·^+^ formed by interaction with SO_4_^−^ [42]. Subsequently, the sulfonamide bond is cleaved by reactive oxygen species to produce monohydroxylated sulfanilic acid and 3-amino-5-methyl-isoxazole [43,44]. In pathway B, cleavage of the S–N bond, which leads to the hydrolyzation of SMX into C_6_H_8_NO_2_S and C_5_H_7_N_2_O, respectively. In pathway C, the H were replaced by the O forming the 5-(4-methoxy phenyl)-1 and 4-oxadiazoles-2-mercaptan [45].

## 4. Conclusions

This study evaluated the photo-Fenton ceramic membrane filtration toward the removal and degradation of SMX as a model recalcitrant micropollutant. The removal of SMX in feed water as well as the raw toilet wastewater in the presence of H_2_O_2_ and UV irradiation was over 80%. The background constituents such as TOC or ammonia in raw toilet wastewater did not appear to affect the photodegradation of SMX. The TOC removal and degradation by-products analysis revealed three possible SMX degradation pathways with four main oxidation intermediates. The research findings laid groundwork toward the application of photo-Fenton reactive membranes for such as wastewater treatment and water reuse.

## Figures and Tables

**Table 1:** Major water quality parameters of the toilet wastewater before/after prefiltration.

Parameters	pH	TOC (mg·L^−1^)	SS (mg·L^−1^)	NH_4_^+^-N (mg·L^−1^)	TP (mg·L^−1^)
Raw	6.94 ± 0.01	1712 ± 18	983 ± 9	1218 ± 16	66 ± 3
Pre-filtered	6.94 ± 0.01	1524 ± 21	N.A.	1168 ± 13	62 ± 2

TOC: total organic carbon; SS: Suspended solid; NH_4_^+^-N: ammonia nitrogen; TP: total phosphors.

**Table 2:** First-order degradation kinetics rate constants of SMX under different experimental conditions in Figure 2a with the uncoated and coated ceramic membranes.

Membrane Type	Reaction Type	First-Order Kinetic Rate Constant (min^−1^)	*R* ^2^
No membrane	UV only	0.0126	0.9654
	H_2_O_2_ only	0.0005	0.9346
	UV + H_2_O_2_	0.0411	0.9436
Uncoated membrane	No UV or H_2_O_2_	0.0001	0.9855
	UV only	0.0213	0.9781
	H_2_O_2_ only	0.0005	0.9345
	UV + H_2_O_2_	0.0928	0.9776
Coated membrane	No UV or H_2_O_2_	0.0001	0.9674
	UV only	0.1435	0.9532
	H_2_O_2_ only	0.0005	0.9762
	UV + H_2_O_2_	1.0031	0.9683

## Supplementary Materials

The following are available online at https://www.mdpi.com/2079-4991/10/1/180/s1, Figure S1: Schematics of the batch experiment of Photo-Fenton degradation. Figure S2: Schematic of the filtration unit with the Photo-Fenton ceramic membrane. Figure S3: The pure water fluxes of the pristine and the catalyst-coated ceramic membranes. Figure S4: (a) The degradation kinetics of SMX and (b) The apparent quantum yield (QY) under different UV_254_ irradiation intensities. Initial SMX concentration: 20 mg·L^−1^; H_2_O_2_ concentration was 10 mmol·L^−1^; and the catalyst on the ceramic membrane was 2 µg·g^−1^.
