# Supplementary material for: Enhanced Degradation of Sulfamethoxazole (SMX) in Toilet Wastewater by Photo-Fenton Reactive Membrane Filtration
Source: Nanomaterials (Basel). 2020 Jan 20;10(1):180. doi: 10.3390/nano10010180 (PMC7023487; doi:10.3390/nano10010180)
Supplement: Supplementary file 1 [file nanomaterials-10-00180-s001.pdf]

Supporting Information

For

Enhanced Degradation of Sulfamethoxazole (SMX) in Toilet Wastewater by Photo-Fenton Reactive Membrane Filtration

Shaobin Sun[1,2], Hong Yao[1*], Xinyang Li[1], Shihai Deng[1,3], Shenlong Zhao[1,5], Wen Zhang[1,2,4]

[1] Beijing International Scientific and Technological Cooperation Base of Water Pollution Control Techniques for Antibiotics and Resistance Genes, Beijing Key Laboratory of Aqueous Typical Pollutants Control and Water Quality Safeguard, Department of municipal and environmental Engineering, School of civil engineering, Beijing Jiaotong University, Beijing, 100044, PR China

[2] John A. Reif, Jr. Department of Civil and Environmental Engineering, New Jersey Institute of Technology, 07102, the US

[3] Department of Civil and Environmental Engineering, National University of Singapore, 10 Kent Ridge Crescent, 119260, Singapore

[4] School of Environmental and Municipal Engineering, Qingdao University of Technology, Qingdao, 266033, China

[5] School of Chemical and Biomolecular Engineering, The University of Sydney, Sydney, NSW 2006, Australia.

[*] Corresponding authors: Hong Yao (yaohongts@163.com)

S1. Experimental setup

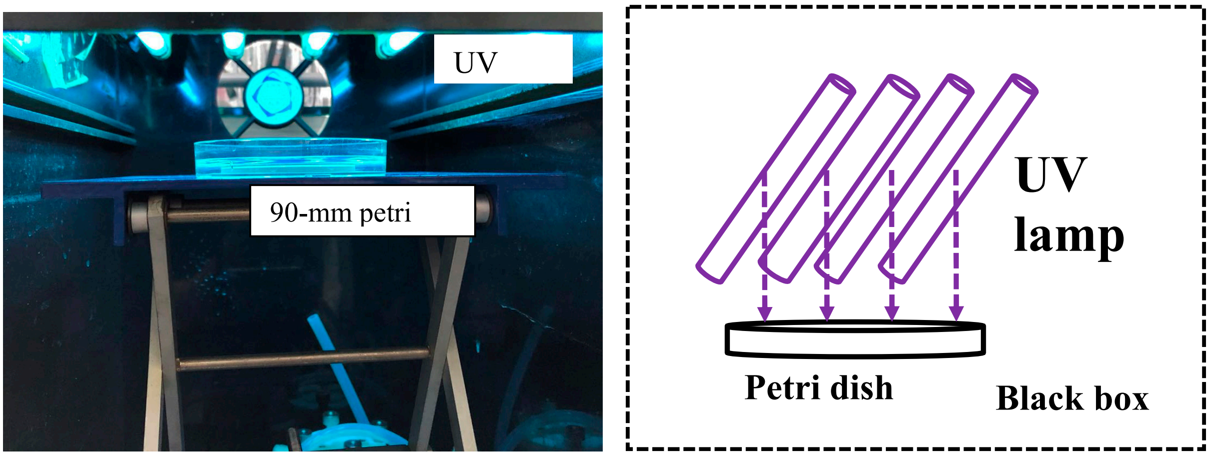

Fig. S1 Schematics of the batch experiment of Photo-Fenton degradation.

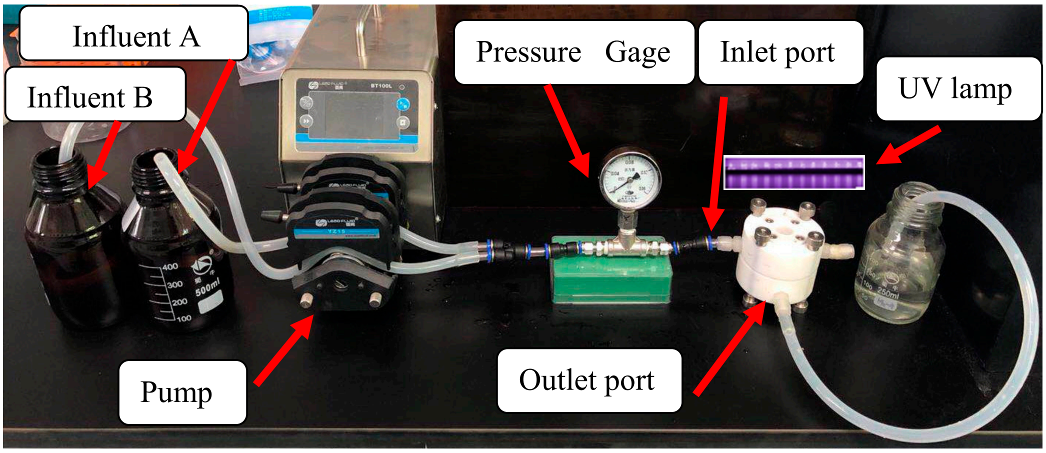

Fig. S2 Schematic of the filtration unit with the Photo-Fenton ceramic membrane.

S2. Detection methods

1. Detection and quantification of SMX

The SMX concentration was measured by a high-performance liquid chromatography (HPLC, WATERS e2695, USA) equipped with an Inertsil ODS-3 column (3 μm, 4.6 mm × 150 mm length) and a photodiode array detector (WATERS 2998). A mixture of DI water (pH 2.5) and acetonitrile (75:25, v/v) was adopted as the mobile phase with a flow rate of 1.0 mL min^{-1}. The determined wavelength was set at 270 nm for SMX.[1]

2. Detection and quantification of hydroxyl radical

The concentrations of pCBA in the reaction medium were analyzed with a same HPLC equipped with an Agilent Zorbax RX-C 18 column and a diode-array UV detector (237 nm). pCBA was eluted using a mixture of methanol (HPLC grade, Sigma-Aldrich) and DI water at 65:35 (v/v).2

3. TOC measurement

Five mL of liquid samples was taken into the tube and diluted to 10 mL. Then, one drop concentrated sulfuric acid was added to the tube and 0.25 mL of this liquid sample was injected to the analyzer. The temperature was set at 800℃; the pressure was 950~1050 MPa and each sample was detected by three times.

4. Mass spectral analysis

Chromatographic separation was achieved in a Venusil 18 column (100 mm × 2.1 mm; 3 μm particle diameter; Bona Angela Technologies, Wilmington, DE, USA). The flow rate adopted was 0.2 mL min−1, and a volume of 5μL was injected for all standard and sample solutions. 3

S3. Determination of membrane permeability

The permeate flux, commonly expressed in units of liters per m2 of membrane per hour (L m-2 h-1, LMH), can be calculated by the Darcy's equation in Eq. 1:

$$J_w = \frac{V}{At} = \frac{TMP}{u(R_m + R_f)} = \frac{P_F - P_P}{u(R_m + R_f)} \quad (1)$$

where J is the permeate flux (LMH), V is the permeate volume (L), A is the effective surface area of the membranes (m2) and t is the time of the permeate collection (h), ΔP is the trans-membrane pressures (TMP), which is the difference of the hydraulic pressure in the feed stream (PF) and the hydraulic pressure in permeate stream (Pp). PF was monitored by a pressure gauge (PEM-LF SERIES, WINTERS), while Pp was equal to the atmospheric pressure. μ is the dynamic viscosity of water at 25 °C (0.8937 × 10−3 N·s·m-2), Rm is the background membrane resistance and Rf is the fouling layer resistance, which contribute to the total membrane hydraulic resistance (for clean water tests, Rf =0).

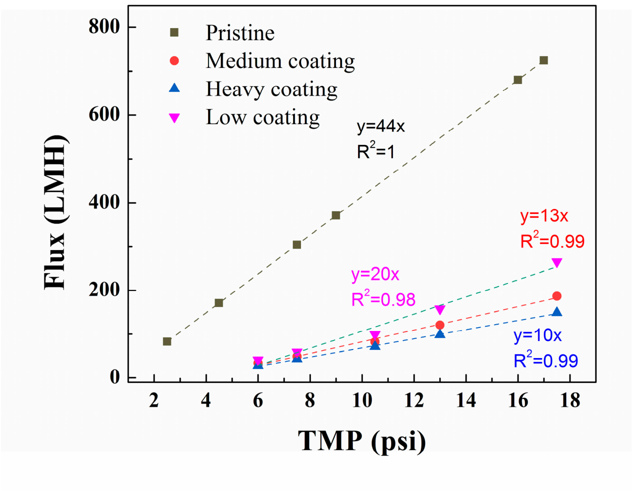

Fig. S3. The pure water fluxes of the pristine and the catalyst-coated ceramic membranes.

S4. Evaluation of the impacts of UV intensity on SMX photodegradation

Fig. S4 shows the degradation kinetics of SMX at different UV intensities from 100 to 400 μW·cm-2. The degradation rate of SMX increased monotonously with the increase of the UV intensity, suggesting that the degradation of SMX was primarily controlled by photo irradiation. However, when the UV intensity increased from 200 to 300 μW·cm-2, the degradation rate did not increase as significantly as expected as the degradation kinetics become limited by the reaction sites, whereas the UV irradiation may have reached the absorption capacity of the coated catalysts. To verify this speculation, we calculated the apparent quantum yield (AQY) for the SMX degradation under four different levels of UV irradiation:

$$\mathrm{AQY}=\frac{\#\ \text{of SMX degraded per time}}{\#\ \text{of UV photons per time}} \tag{1}$$

Since the degradation rate varied with the reaction time, the stable degradation rates around 5 min were used in the calculation. The UV photon flux (#photon·cm-2·s-1) under different UV intensities (μW·cm-2) are computed by the Einstein's equation and then converted to the rate of applied photons (#photon·s-1) by multiplying the UV-irradiated area as mentioned above. Fig. S4b shows that the highest AQY was approximately 27%, highlighting the high efficiency of photo-Fenton degradation reactions. The AQY gradually declined when increasing the UV intensity from 100 to 400 μW·cm-2. This result indicates that the UV irradiation applied to the membrane surface should be optimized based on the available reaction sites of the coated catalyst. This result also implies that the applied UV intensity at 200 μW·cm-2 or less in our experiment should reach a reasonably high AQY.

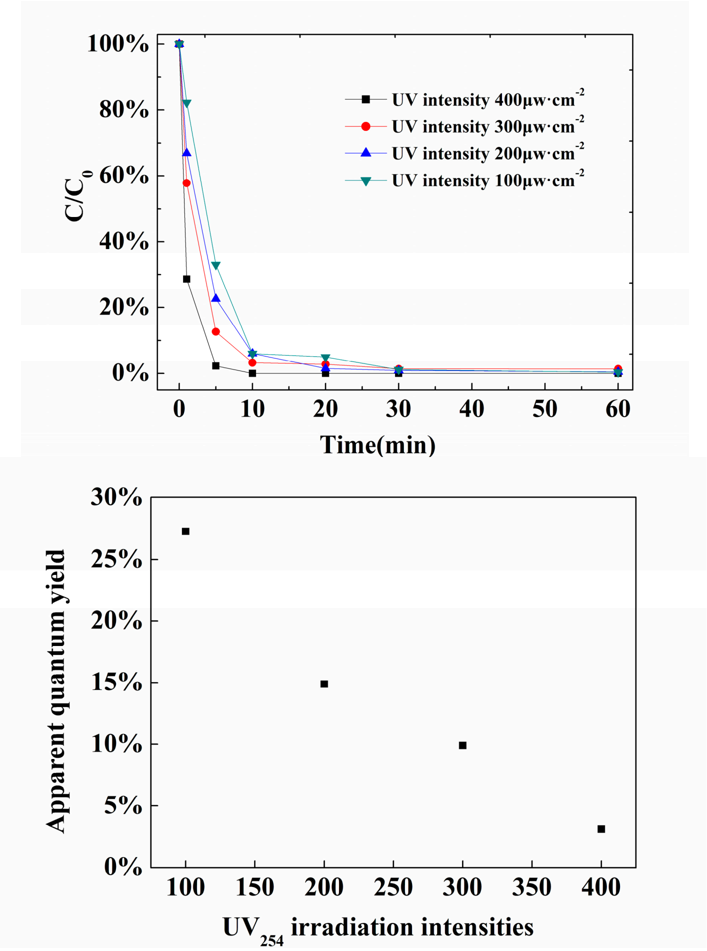

Fig. S4. (a) The degradation kinetics of SMX and (b) The apparent quantum yield (QY) under different UV_{254} irradiation intensities. Initial SMX concentration: 20 mg·L^{-1}; H_2O_2 concentration was 10 mmol·L^{-1}; and the catalyst on the ceramic membrane was 2 μg·g^{-1}.

References

1. Wang, J.; Zhou, T.; Mao, J.; Wu, X., Comparative study of sulfamethazine degradation in visible light induced photo-Fenton and photo-Fenton-like systems. *Journal of Environmental Chemical Engineering* **2015,** *3*, 2393-2400.
2. Jeong, J.; Kim, C.; Yoon, J., The effect of electrode material on the generation of oxidants and microbial inactivation in the electrochemical disinfection processes. *Water Research* **2009,** *43*, 895-901.
3. Seraglio, S. K. T.; Valese, A. C.; Daguer, H.; Bergamo, G.; Azevedo, M. S.; Gonzaga, L. V.; Fett, R.; Costa, A. C. O., Development and validation of a LC-ESI-MS/MS method for the determination of phenolic compounds in honeydew honeys with the diluted-and-shoot approach. *Food Research International* **2016,** *87*, 60-67.
4. Neamu, M.; Catrinescu, C.; Kettrup, A., Effect of dealumination of iron(III)—exchanged Y zeolites on oxidation of Reactive Yellow 84 azo dye in the presence of hydrogen peroxide. *Applied Catalysis B: Environmental* **2004,** *51*, 149-157.
5. Sun, S.; Yao, H.; Fu, W.; Hua, L.; Zhang, G.; Zhang, W., Reactive Photo-Fenton ceramic membranes: Synthesis, characterization and antifouling performance. *Water Research* **2018,** *144*, 690-698.
6. Monash, P.; Pugazhenthi, G., Effect of TiO 2 addition on the fabrication of ceramic membrane supports: A study on the separation of oil droplets and bovine serum albumin (BSA) from its solution. *Desalination* **2011,** *279*, 104-114.
7. Lee, J.; Jeong, S.; Ye, Y.; Chen, V.; Vigneswaran, S.; Leiknes, T. O.; Liu, Z., Protein fouling in carbon nanotubes enhanced ultrafiltration membrane: Fouling mechanism as a function of pH and ionic strength. *Separation & Purification Technology* **2017,** *176*, 323-334.
